# Sensitivity of mice to lipopolysaccharide is increased by a high saturated fat and cholesterol diet

**DOI:** 10.1186/1476-9255-4-22

**Published:** 2007-11-12

**Authors:** Hong Huang, Tongzheng Liu, Jane L Rose, Rachel L Stevens, Dale G Hoyt

**Affiliations:** 1 Division of Pharmacology, The Ohio State University College of Pharmacy, Columbus, OH, 43210, USA; 2 The Dorothy M. Davis Heart and Lung Research Institute, Columbus, OH, 43210, USA; 3 Center for Cardiovascular Medicine, Columbus Children's Research Institute, Columbus, OH, 43205, USA

## Abstract

**Background:**

It was hypothesized that a pro-atherogenic, high saturated fat and cholesterol diet (HCD) would increase the inflammatory response to *E. coli *endotoxin (LPS) and increase its concentration in plasma after administration to mice.

**Methods:**

C57Bl/6 mice were fed a HCD or a control diet (CD) for 4 weeks, and then treated with saline, 0.5, 1 or 2 mg LPS/kg, ip. Liver injury (alanine:2-oxoglutarate aminotransferase and aspartate aminotransferase, collagen staining), circulating cytokines (tumor necrosis factor-α, interleukin-6 and interferon-γ), factors that can bind LPS (serum amyloid A, apolipoprotein A1, LPS binding protein, and CD14), and plasma levels of LPS were measured. The hepatic response was assessed by measuring vascular cell adhesion molecule (VCAM)-1, inducible nitric oxide synthase (iNOS) and signal transducer and activator of transcription-1 proteins, and VCAM-1 and iNOS mRNAs. Hepatic mRNA encoding the LPS receptor, Toll like receptor 4, was also determined.

**Results:**

Two mg LPS/kg killed 100% of mice fed HCD within 5 d, while no mice fed CD died. All mice treated with 0 to 1 mg LPS/kg survived 24 h. HCD increased plasma alanine:2-oxoglutarate aminotransferase and aspartate aminotransferase, and the enzymes were increased more by LPS in HCD than CD mice. Induction of plasma tumor necrosis factor-α, interleukin-6, and interferon-γ by LPS was greater with HCD than CD. Hepatic VCAM-1 and iNOS protein and mRNA were induced by LPS more in mice fed HCD than CD. Tyrosine phosphorylation of signal transducer and activator of transcription-1 caused by LPS was prolonged in HCD compared with CD mice. Despite the hepatic effects of HCD, diet had no effect on the LPS plasma concentration-time profile. HCD alone did not affect circulating levels of plasma apolipoprotein A1 or LPS binding protein. However, plasma concentrations of serum amyloid A and CD14, and hepatic toll-like receptor-4 mRNA were increased in mice fed HCD.

**Conclusion:**

HCD increased the sensitivity of mice to LPS without affecting its plasma level. Although increased serum amyloid A and CD14 in the circulation may inhibit LPS actions, their overexpression, along with hepatic toll-like receptor-4 or other factors, may contribute to the heightened sensitivity to LPS.

## Background

Sepsis is a complex syndrome that results from the host response to infection. Systemic effects of Gram-negative sepsis are mediated in large part by lipopolysaccharide (LPS), which causes tissue injury and inflammation. During bacterial sepsis, as opposed to focal infection or local inflammation, a major organ that responds to LPS is the liver [1,2]. LPS induces hepatic production of acute phase proteins, such as C-reactive protein, serum amyloid A (SAA), CD14, and LPS binding protein (LBP), which may actually function to restrict LPS action [3-6].

LPS causes endothelial activation with up-regulation of adhesion molecules, and promotes the release cytokines, including tumor necrosis factor-α (TNFα), interleukin-6 (IL-6) and interferon-γ (IFNγ), which typically increase in sequence [7-9]. LPS, directly and via cytokines, activates transcription of pro-inflammatory genes such as inducible nitric oxide synthase (iNOS), and vascular cell adhesion molecule-1 (VCAM-1). This results in production of nitric oxide that may contribute to shock and other events, while endothelial VCAM-1 mediates sequestration of leukocytes in tissues [10]. The induction of iNOS and VCAM-1 is driven by the activation of several transcription factors, including nuclear factor kappa B (NFkB), activating protein-1 (AP-1) and signal transducer and activator of transcription-1 (STAT1) [11-14].

Genetic and extrinsic factors affect the response to LPS. One extrinsic factor is diet. High dietary cholesterol increased susceptibility to various viral and bacterial infections [15-19]. Dietary cholesterol increased serum amyloid A, histocompatibility class II, TNFα and other inflammatory molecules in response to LPS [20-24], and high fat diet induced histocompatibility class II [25]. Toll-like Receptor 4 (TLR4), which mediates many effects of LPS, was expressed in atherosclerotic lesions [26,27], and aortas of TLR4 knockout mice had reduced inflammatory activation in response to a high fat diet [28], suggesting a role for this endotoxin receptor in these diet-related effects. Dietary fat and cholesterol could also increase responses to LPS by increasing its blood levels after exposure. Given the inflammatory effects of atherogenic diets, and the role of endotoxins in acute and chronic disease [7,22], we hypothesized that a high saturated fat and cholesterol diet (HCD) would increase effects of LPS and its plasma concentration after administration to mice.

## Methods

**Figure 1:**
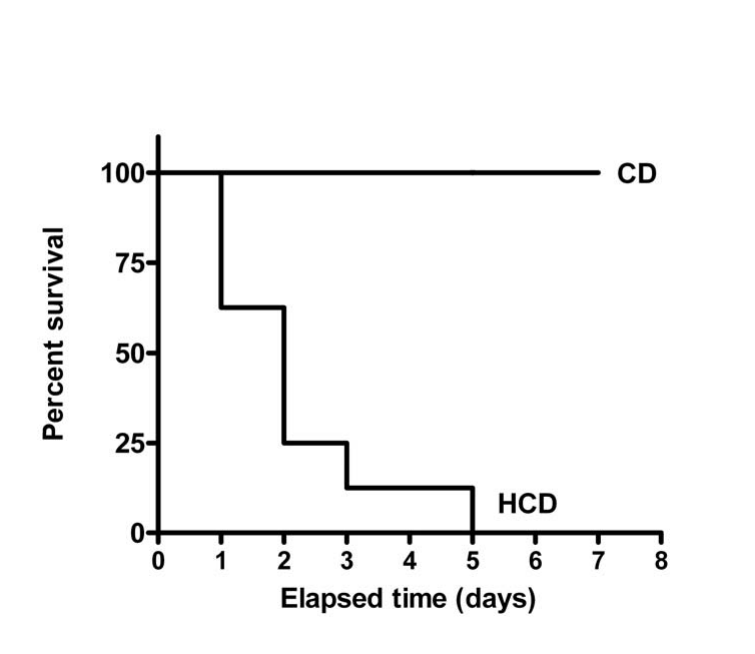
The effect of LPS on survival of mice. C57Bl/6 mice (8 per group), were fed HCD or CD for 4 wk, and then treated with a single dose of LPS (2 mg/kg, ip). The median survival time for mice fed HCD was 2 d and the curves differed significantly (p < 0.0001).

Female C57BL/6 mice, 3–4 wk old, were purchased from Jackson laboratories, Bar Harbor, ME. Mice were randomly fed the HCD or control diet (CD) (Research Diets, Inc., New Brunswick, NJ, Table 1) for 4 wk, when cholesterol reached a steady level in HCD mice (data not shown). Mice were treated with an intraperitoneal (ip) injection of *E. coli *LPS, serotype 0111:B4 (Sigma-Aldrich, Inc., St. Louis, MO) in sterile saline solution after 4 wk feeding. The protocol was carried out under approval of the Ohio State University Animal Care and Use Committee. In the first experiment, 8 mice per group were treated with 0 or 2 mg LPS/kg, ip. With the unexpected lethality in HCD mice (figure 1), independent groups fed CD or HCD were subsequently treated with of 0, 0.5, or 1.0 LPS mg/kg. For plasma LPS determinations, pilot experiments indicated that serial sampling of blood from mice produced samples variably contaminated with LPS. Therefore, a single sample of blood was taken from the right ventricle of 5 individual mice in each group. Plasma was recovered and stored at -80°C and livers were removed and frozen in liquid nitrogen.

**Table 1:** Composition of the purified HCD and CD

	HCD (D12336)^1^		CD (D12337)^1^	
Ingredient	g/kg of Diet	kJ/g of Diet	g/kg of Diet	kJ/g of Diet
Casein, 30 Mesh	75	1.26	67	1.12
Soy Protein	130	2.18	116	1.94
DL-Methionine	2	0.03	1.8	0.03
Corn Starch	275	4.60	466	7.81
Maltodextrin 10	150	2.51	134	2.24
Sucrose	30	0.50	27	0.45
Cellulose, BW200	90	0	80	0
Soybean Oil	50	1.88	45	1.68
Cocoa Butter	75	2.3	0	0
Coconut Oil, 76	35	1.32	0	0
Mineral Mix S10001 [60]	35	0	31.2	0
Calcium Carbonate	5.5	0	4.9	0
Sodium Chloride	8	0	7.1	0
Potassium Citrate	10	0	8.9	0
Vitamin Mix V10001 [60]	10	0.17	8.9	0.15
Choline Bitartrate	2	0	1.8	0
Cholesterol, USP	12.5	0	0.27	0
Sodium Cholic Acid	5	0	0	0
FD&C Red Dye #40	0.10	0	0	0
FD&C Blue Dye #40	0	0	0.09	0
Total	1000	16.8	1000	15.4

^1^Product Number from Research Diets, Inc. is indicated in parentheses.

Plasma cholesterol was measured with Infinity Cholesterol Reagents (Sigma-Aldrich, Inc., St. Louis, MO). Plasma alanine:2-oxoglutarate aminotransferase (ALT), aspartate aminotransferase (AST) were measured with a kinetic assay [29], using reagents from Thermo Trace, Louisville, CO. Limulus amoebocyte lysate chromogenic endpoint assay (Hycult Biotechnology b. v., Norwood, MA) was used for LPS detection [30]. Absorbance was read at 405 nm. To exclude the possibility that lipid levels would affect the sensitivity of the assay, various standard curves with reconstituted LPS diluted in plasma collected from untreated CD or HCD mice. There was no statistical difference between these curves (data not shown).

Plasma samples were diluted with endotoxin-free water and tested with ELISA kits for TNFα, IL-6, and IFNγ (BD Bioscience, Pharmagen) and SAA (BioSource International, Camarillo, CA). Wells of plates were coated with primary antibody standards or samples were incubated in the wells, treated with avidin-horseradish peroxidase. 3, 3', 5, 5'-Tetramethyl benzidine was added to develop color. Absorbance was read at 450 nm after stopping with 1 mol/L H_3_PO_4_.

Five ul plasma were also mixed with denaturing sample buffer [11], heated 10 min at 95 degrees, and subjected to SDS-PAGE and western blotting with anti-mouse apolipoprotein A1 (Abcam, Inc., Cambridge, MA), anti-mouse CD14 (BD Pharmingen, San Diego, CA) or anti-mouse LBP (Cell Sciences, Inc., Canton, MA). Ten mg of liver tissue were lysed in 100 μL RIPA buffer (1% TritonX-100, 50 mM Tris, pH 7.5, 150 mmol/L NaCl, 5 mmol/L EDTA, 0.5 mmol/L Na_3_VO_4_, 50 mmol/L NaF, 10 mg aprotinin/L, 10 mg leupeptin/L, 10 mg pepstatin A/L, and 1 mmol/L PMSF) and sonicated. Protein concentrations were determined [31], and 10 μg liver protein was subjected to western blotting [11]. Antibodies used were rabbit anti-inducible nitric oxide synthase (iNOS) (Transduction Laboratories, Lexington, KY), goat anti-mouse vascular cell adhesion molecule-1 (VCAM-1) (Santa Cruz Biotechnology, Inc. Santa Cruz, CA), rabbit anti-signal transducer and activator of transcription 1, total (STAT1), and anti-phospho-STAT1 (Y701) (Cell Signaling Technology, Inc. Beverly, MA). Horseradish peroxidase-conjugated goat anti-mouse, goat anti-rat, goat anti-rabbit, rabbit anti-goat secondary antibodies (Jackson ImmunoResearch Laboratories, Inc. West Grove, PA) were used to detect labeled proteins with enhanced chemiluminescence and X-ray film exposure. Films were digitally scanned and integrated signal intensity of bands was calculated by image analysis (NIH Image J).

Approximately 50 mg of liver was homogenized in 1 mL of Trizol reagent, and total cellular RNA was extracted, reverse transcribed into cDNA and subjected to polymerase chain reaction as described previously (primers, reagents and enzymes were from Invitrogen Corporation, Grand Island, NY) [11]. The primers used in this study were: iNOS [GenBank: NM_010927], sense: 5'-CCT GGA CAA GCT GCA TAT GA-3'; antisense: 5'-GCT GTG TGG TGG TCC ATG AT-3'; VCAM-1 [GenBank: NM_011693], sense: 5'-GCG CTG TGA CCT GTC TGC AA-3' and antisense: 5'-GGT GTA CGG CCA TCC ACA G-3'; TLR4 [GenBank: AF185285], sense: 5'-GCT TAC ACC ACC TCT CAA ACT TGA T-3', antisense: 5'-ATT ACC TCT TAG AGT CAG TTC ATG G-3'; β-actin [GenBank: X03672], sense: 5'-ATG GAT GAC GAT ATC GCT-3', antisense: 5'-ATG AGG TAG TCT GCT AGG T-3'. The polymerase chain reaction conditions included an initial denaturation at 94°C for 5 min, followed by a cycle of denaturation (94°C/1 min), annealing (1 min at 60°C for iNOS and β-actin, or at 55°C for VCAM-1 and TLR4), and extension (72°C/1 min). Each sample was subjected to 35 cycles followed by a final extension (72°C for 10 min). Equal amounts of RNA, determined by absorbance at 260 nm, were reverse transcribed. Polymerase chain reaction products were separated and visualized on a 1.0% agarose ethidium bromide stained gel. Band intensity was assessed from digital images with analysis software (UVP-Labworks Analysis), normalized to β-actin expression in each sample. The densitometric ratio of target mRNA signal to β-actin signal was calculated.

Hepatic fibrosis was evaluated in formalin-fixed, paraffin-embedded tissues using Masson's trichrome stain (Sigma-Aldrich, Inc., St. Louis, MO) [32]. Images were captured using a 20× objective on a standard upright microscope (Olympus, Melville, NY) and a digital camera (Diagnostic Instruments, Sterling Heights, MI). Five images were captured per section from 5 mice fed CD or HCD. Blue collagen staining was defined, and the extent of fibrosis, as a percentage of total tissue area in each image, was calculated using Image Pro Plus image analysis software (Media Cybernetics, Silver Springs, MD).

Survival was analyzed by the Mantel-Haenszel test. Other data were analyzed by Student's t-test or by ANOVA with Bonferroni adjustment for multiple comparisons of independent groups [33]. A p-value less than 0.05 was considered significant.

## Results

Plasma cholesterol was increased by 4 wk feeding HCD in comparison with CD (means ± SE were 3.3 ± 0.02 in CD mice and 5.3 ± 0.02 mmol/L in HCD mice, p < 0.0001). Body weights did not differ between mice fed the CD (23.37 ± 0.04 g) and HCD (23.42 ± 0.04 g). Food intake over 4 wk did not differ between the groups (1.9 ± 0.19 vs. 1.8 ± 0.17 g/mouse/d for CD and HCD), and energy intake was not different (30.5 ± 2.8 and 31.9 ± 2.9 kJ/mouse/d in mice fed CD and HCD respectively). These results are similar to those of others using similar diets with C57Bl/6 mice [34]. Liver weights were significantly increased in HCD as compared with CD mice: liver to body weight ratio was 4.15 ± 0.005% in mice fed the CD and 6.37 ± 0.012% in mice fed the HCD (p < 0.0001).

**Figure 2:**
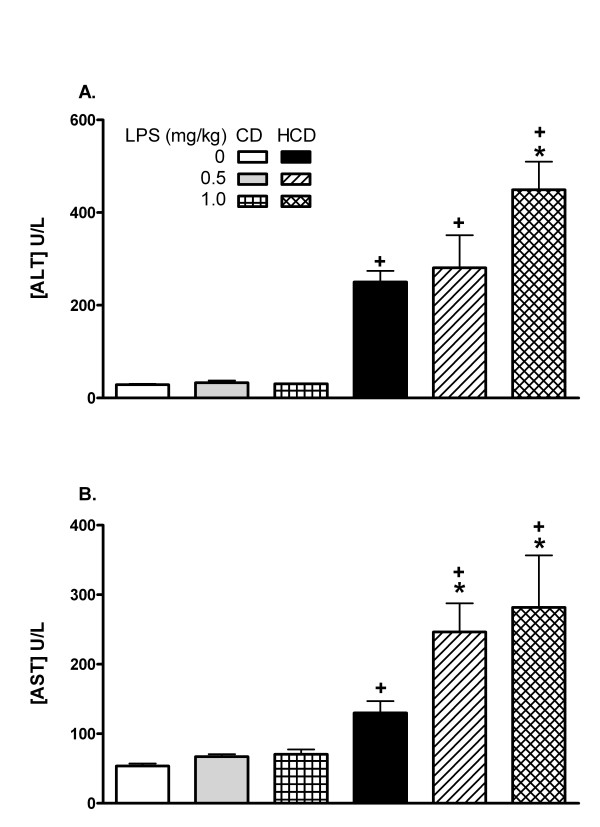
The effect of diet on plasma ALT and AST. CD or HCD mice received a single ip injection of 0, 0.5, or 1.0 mg LPS/kg and blood was sampled after 12 h. Values are mean + SE of 5 mice per group. *: p < 0.05 for comparison with 0 mg LPS/kg. +:p < 0.05 for comparison with CD mice treated with the same dose of LPS.

C57BL/6 mice fed HCD were extremely sensitive to LPS: after a single ip dose of 2 mg/kg none of the mice survived for 5 d, compared with a 100% survival in the CD group (Figure 1). The median survival time for mice fed HCD was 2 d and the curves differed significantly (p < 0.0001). Therefore, lower doses of LPS (0.5 to 1 mg/kg) were used for subsequent experiments. Plasma ALT and AST activities were elevated in saline-treated mice fed the HCD in comparison with CD, and were further elevated only in HCD mice 12 h after LPS treatment (figure 2). The extent of fibrosis, indicated by the relative area of collagen staining in liver sections, was not affected by diet (relative areas were 0.057 ± 0.017 in CD and 0.076 ± 0.054 in HCD sections).

**Figure 3:**
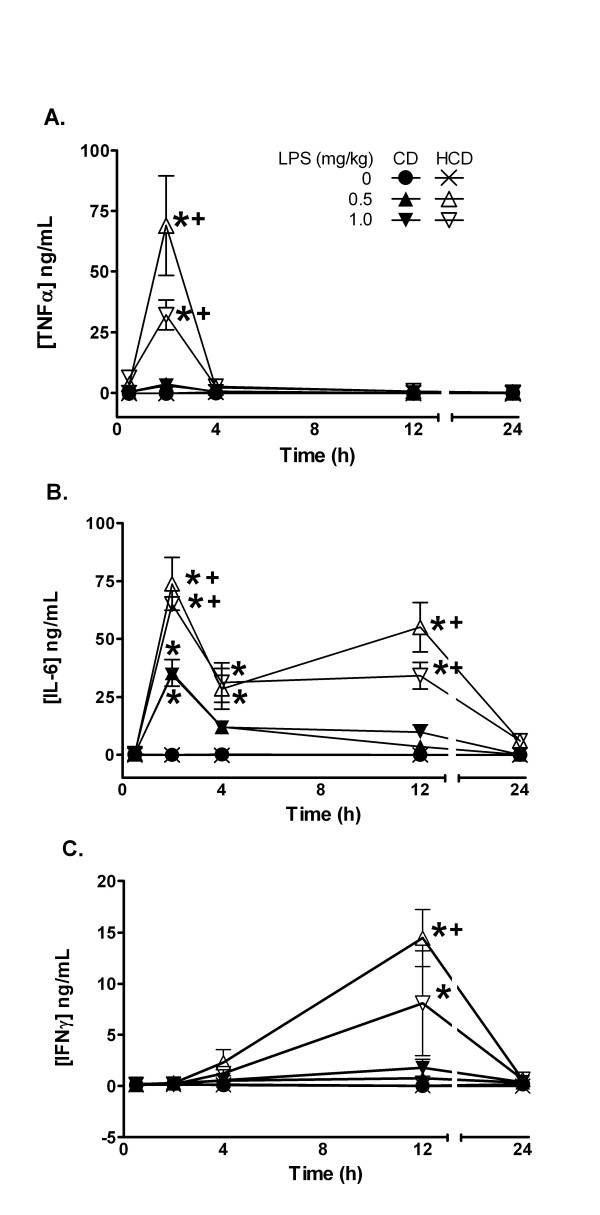
The effect of diet and LPS on plasma levels of pro-inflammatory TNFα (A), IL-6 (B), and IFNγ (C). Plasma levels were measured 0.5, 2, 4, 12, or 24 h after treatment. Values are the mean ± SE of 5 mice per group. *: p < 0.05 for comparison with 0 mg LPS/kg. +: p < 0.05 for comparison with CD treated with the same dose of LPS.

The effect of HCD on plasma cytokines that mediate some effects of LPS was determined. Plasma TNFα was elevated only in CD mice treated with 1 mg LPS/kg at 2 h, while increases were significantly greater in LPS-treated HCD mice (Figure 3A). IL-6 and IFNγ were also significantly increased by LPS, and the increases were greater in HCD mice compared with CD (Figure 3B and 3C). In particular, IL-6 remained elevated for at least 12 h in HCD mice and the typically delayed increase in IFNγ was observed 12 h after LPS only in HCD mice.

**Figure 4:**
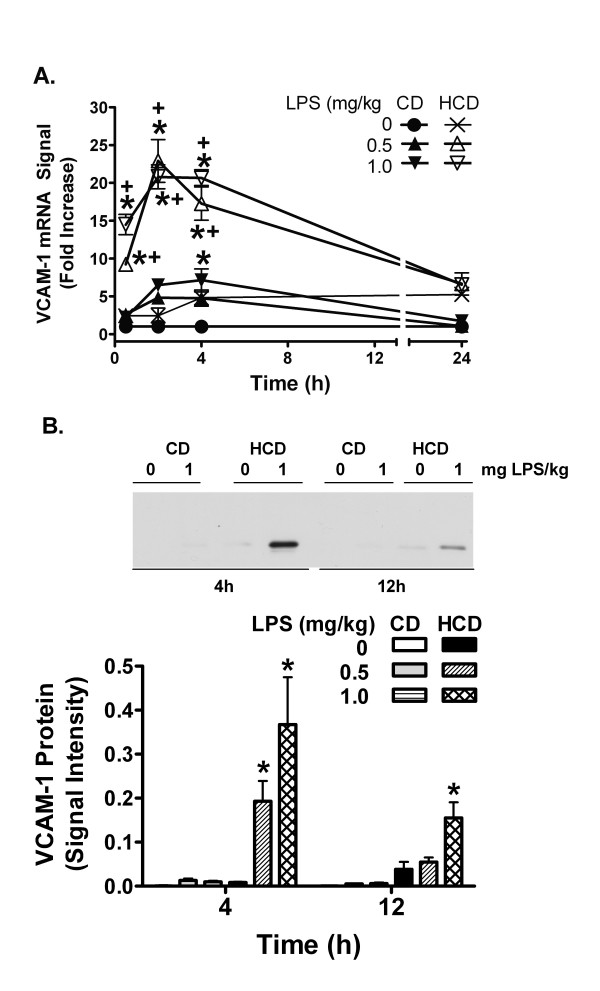
The effect of diet and LPS on hepatic VCAM-1 expression. mRNA levels (A) were measured with RT-PCR. Densitometric intensity of images of ethidium bromide-stained agarose gels were normalized to β-actin RT-PCR signal. Induction of VCAM-1 RT-PCR signal ratios (n = 5 in each group) are expressed as fold increase compared to vehicle-treated CD mice, represented by 1 on the y-axis. Values are expressed mean ± SE of 5 mice are the fold increases in VCAM-1/β-actin mRNA signal ratio relative CD mice treated with 0 mg LPS/ml (represented by 1 on the y-axis). The level of VCAM-1 protein (B) was measured by western blotting 4 and 12 h after treatment (representative images and image analysis). Values are the mean + SE of total integrated signal intensity obtained from densitometry for 5 mice in each group. *: p < 0.05 for comparison with 0 mg LPS/kg. +: p < 0.05 for comparison with CD treated with the same dose of LPS.

**Figure 5:**
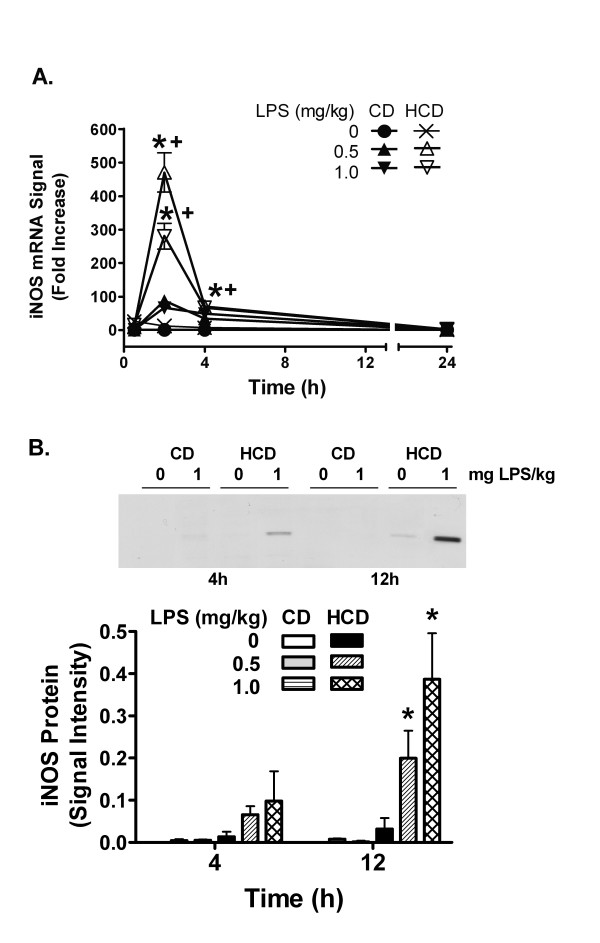
The effect of diet and LPS on hepatic iNOS expression. iNOS mRNA (A) was assessed as in figure 4. Values are expressed mean ± SE of 5 mice are the fold increases in iNOS/β-actin mRNA signal ratio relative CD mice treated with 0 mg LPS/ml, represented by 1 on the y-axis. The level of iNOS protein (B) was measured by western blotting 4 and 12 h after treatment (representative images and image analysis). Values are the mean + SE of total integrated signal intensity obtained from densitometry for 5 mice in each group. *: p < 0.05 for comparison with 0 mg LPS/kg. +: p < 0.05 for comparison with CD treated with the same dose of LPS.

Hepatic VCAM-1 mRNA was induced 0.5 h after LPS injection with respect to the saline-treated mice in both diet groups, but was increased more in the HCD mice (Figure 4A). Induction reached a peak at 2–4 h and declined over 24 h. While VCAM-1 protein was not increased by LPS in CD mice, it was significantly elevated in HCD mice 4 h after administration of 0.5 or 1 mg LPS/kg, and remained elevated 12 h after 1 mg LPS/kg (Figure 4B). The iNOS mRNA signal was increased by LPS in HCD mice with a peak at 2 h (Figure 5A). iNOS protein was increased in HCD mice 12 h after 0.5 and 1 mg LPS/kg, and not in CD mice (Figure 5B).

**Figure 6:**
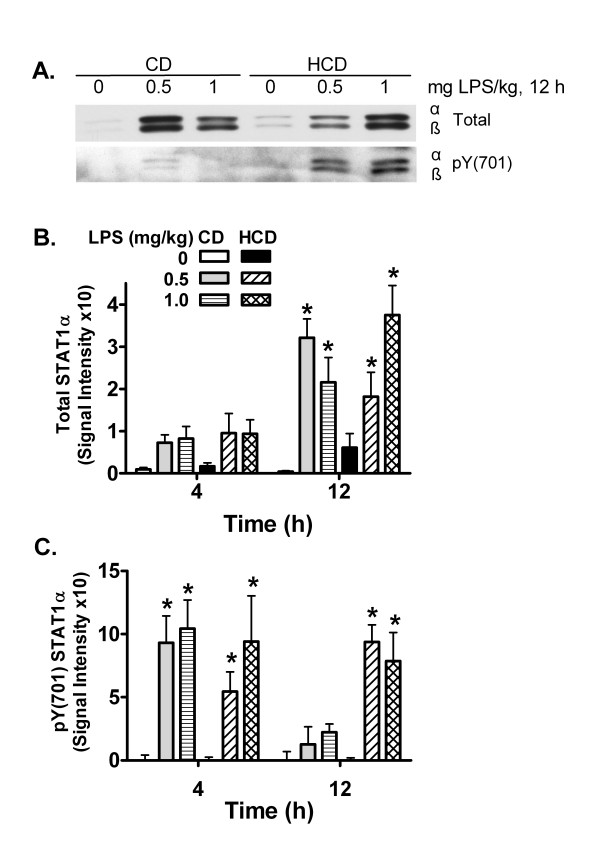
The effect of diet and LPS on STAT1 activation in liver. Total and tyrosine 701 phosphorylated (pY701) STAT1 protein levels in hepatic tissues from CD and HCD mice were measured by western blotting. A representative image from samples 12 h after LPS treatment is shown in panel A. STAT1α and the shorter β form are visible. Densitometric intensity of total and pY701 STAT1α was measured and means + SE of 5 mice fed each diet 4 and 12 h after treatment were calculated (B and C). *: p < 0.05 for comparison with 0 mg LPS/kg.

IL-6 and IFNγ cause inflammatory gene expression in part by activating STAT1 [11,35]. Total levels of STAT1α were increased in HCD and CD mice 12 h after treatment with LPS in comparison with saline (Figure 6A, B). The activated form of STAT1α, phosphorylated at tyrosine 701 (pY701), was increased 4 h after treatment with LPS, in comparison with saline, in both CD and HCD mice. However, elevated phosphorylation was maintained for 12 h in LPS-treated HCD mice, while it had decreased in the CD group (Figure 6A, C).

**Figure 7:**
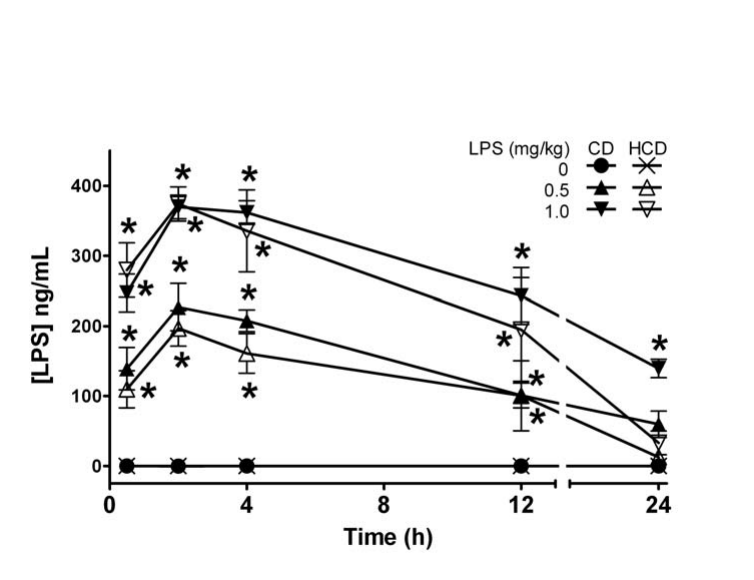
The effect of diet on plasma pharmacokinetics of LPS. CD or HCD mice received a single ip injection of 0, 0.5, or 1.0 mg LPS/kg. Values are mean ± SE of 5 mice per group. *: p < 0.05 for comparison with 0 mg LPS/kg for each diet.

Given the heightened response to LPS, the effect of diet on its plasma levels was determined. LPS was not detectable in blood of saline-treated CD or HCD mice. LPS increased rapidly in plasma after ip injection in both CD and HCD mice (Figure 7). The highest LPS levels occurred at 2 h, and then decreased. There was no difference between mice fed the two diets, except with 1 mg LPS/kg at 24 h, where the level was elevated in CD mice. In another group of mice treated with 1 mg LPS/kg, plasma LPS was undetectable 7 d after treatment (data not shown). The half-life for LPS was approximately 12 h, which is similar to results reported in C3H/St and C3H/HeJ mice [36].

**Figure 8:**
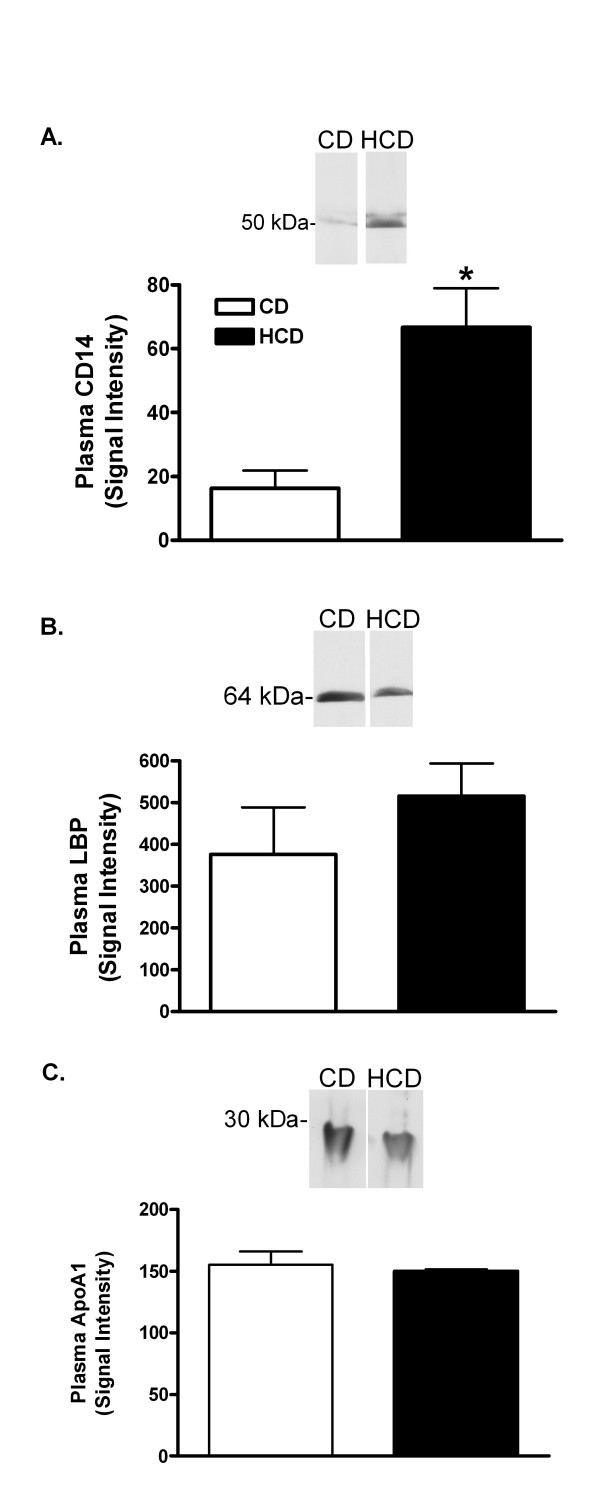
The effect of diet on plasma CD14, ApoA1 and LBP. CD14 (A), LBP (B), and ApoA1 (C) were measured by densitometry after western blotting of plasma samples from mice fed CD or HCD for 4 weeks. Representative blots are shown. Bars depict the mean + SE for 5 mice in each group. *: p < 0.05 for comparison with CD mice.

**Figure 9:**
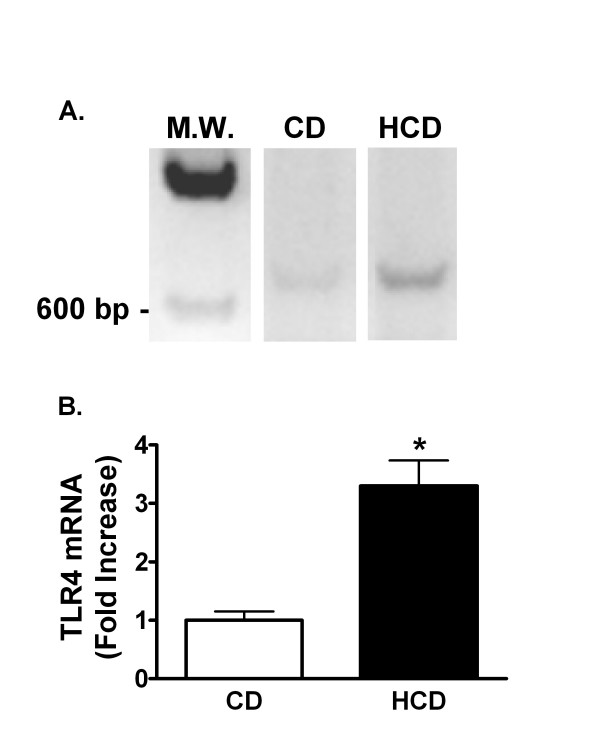
The effect of diet and LPS on hepatic TLR4 mRNA. Representative images of ethidium-stained agarose electrophoresis gels of RT-PCR products of RNA extracted from livers of mice fed CD or HCD for 4 weeks (A). MW is a Lambda HindIII molecular weight marker. Signal intensities were measured as in figure 4A and normalized to the RT-PCR signal for β-actin in each sample (B). Bars depict mean normalized signal ratios + SE for 5 mice fed each diet, relative to the CD group, represented by 1 on the y-axis. *: p < 0.05 for comparison with CD.

The effect of diet on proteins known to bind LPS was investigated. Plasma SAA was increased 84-fold in HCD compared with CD mice (mean ± SE was 1.5 ± 0.9 μg/ml in mice fed the CD, and 126.7 ± 23.9 μg/ml in mice fed HCD, p < 0.0001). Twelve h after treatment with 0.5 mg LPS/kg, SAA was 3912 ± 821 μg/ml in CD and 5424 ± 628 μg/ml in HCD mice. Plasma CD14 w as increased 4.1-fold in HCD compared with CD mice (figure 8A). Plasma LBP and ApoA1, however, were unaffected by diet (Figure 8B, C). Finally, reverse transcription-polymerase chain reaction (RT-PCR) products of TLR4 mRNA were increased in livers of mice fed HCD in comparison with CD mice (Figure 9). Although it would be desirable to assess hepatic TLR4 in mouse liver, several commercially available antibodies identified multiple bands of varying size on western blots of murine samples (not shown).

## Discussion

Feeding HCD for 4 wk raised cholesterol levels and increased the lethal and inflammatory effects of LPS. While there was no evidence of hepatic fibrosis, mild increases in plasma ALT and AST suggested that HCD alone affected the liver. The effect of LPS on these enzymes was also increased in HCD mice. The livers of HCD mice reacted normally to LPS in 2 respects. As detailed later, removal of LPS from plasma was not affected by diet, and, while the plasma SAA was increased by HCD alone, the level to which it was induced by LPS was similar to the induction in CD mice. Further clarification of the role of the liver alteration in sensitivity to LPS is necessary.

Cytokines induced by LPS may mediate its deleterious effects [9]. IFNγ for example causes LPS hypersensitivity [37] and promotes atherosclerosis [38]. Although HCD alone did not elevate plasma TNFα, IL-6 or IFNγ, the higher sensitivity of HCD mice to LPS was paralleled by greater increases in these cytokines in response to LPS in comparison with CD mice (figure 3). The observed pattern of a transient spike in TNFα with increased IL-6, which is known to repress TNFα [39], followed by production of IFNγ, likely from secondarily activated T and B lymphocytes, is a typical response to LPS [9]. Thus, HCD apparently potentiated the cytokine-inducing effects of LPS without qualitatively altering their sequence of appearance. Others found that hypercholesterolemia increased the induction of plasma TNFα, or of aortic interleukin-1 and TNFα mRNA by gram negative endotoxins [22,24]. Interestingly, a high fat diet in rats, or familial hypercholesterolemia in humans, did not increase TNFα production by isolated monocytes or whole blood treated with LPS *in vitro *[40,41]. This suggests that cells in these preparations were not made hyper-responsive to LPS by these conditions. TNFα is constitutively synthesized by Kupffer cells in liver, which rapidly release it after challenge with LPS. While IL-6 is not constitutively expressed, it is rapidly transcribed by these liver macrophages [9]. HCD may increase the sensitivity and/or numbers of circulating leukocytes, and hepatic Kupffer or other cells that could contribute to cytokine production [42,43]. Non-hepatic tissues may also be sensitized *in vivo *[24]. Nevertheless, significant increases in hepatic TLR4 mRNA in untreated HCD mice compared with CD mice support the idea that the liver is made hyper-responsive to LPS by HCD (figure 9).

VCAM-1 and iNOS were measured since they are part of an inflammatory cascade induced by LPS. VCAM-1 mediates localization of various inflammatory cells in tissues. iNOS, on the other hand, can produce large amounts of nitric oxide, a potent vasodilator that may contribute to shock [10]. HCD increased the effect of LPS on hepatic VCAM-1 and iNOS mRNA and protein (Figures 4 and 5), further confirming the pro-inflammatory nature of HCD.

The murine VCAM-1 and iNOS promoters are activated by AP-1, NFkB, and by STAT1 and its secondary target, interferon regulatory factor-1 [12-14,44]. TNFα is a strong activator of AP-1 and NFkB, and IL-6 and IFNγ each cause phosphorylation of STAT1 on tyrosine 701, dimerization, and translocation to the nucleus [11,35,45]. Thus, early induction of VCAM-1 and iNOS mRNA by LPS may result from actions of LPS or the induced TNFα and IL-6 on these transcription factors. The prolonged increase in IL-6 and enhancement of later increases in IFNγ by LPS in HCD mice correlates with the extended duration of STAT1 activation (figure 6). While STAT1 may contribute to acute increases in hepatic iNOS and VCAM-1 mRNA, it cannot be the only factor, since it was similarly activated in both CD and HCD mice 4 h after LPS treatment. However, the extended duration of STAT1 activation in HCD mice may stimulate the delayed expression of other factors. As seen in cardiac myocytes [44], iNOS protein increased well after mRNA levels peaked and returned to baseline (figure 5), suggesting that translation may be increased, or that protein degradation may be reduced in HCD mice at later times after LPS treatment. The role of LPS, TNFα, IL-6, IFNγ, or STAT1 in particular, in the translation or turnover of iNOS and VCAM-1 after HCD remains to be investigated. In any case, inflammatory enhancement by HCD may contribute to a detrimental interaction between diet and LPS *in vivo*.

HCD could increase plasma levels of LPS or its activity. Regardless of diet, however, LPS appeared in the systemic circulation within 30 min and reached similar peaks 2–4 h after ip injection (Figure 7). The entire pharmacokinetic profile of LPS was similar in CD and HCD mice, indicating that the hypersensitivity of HCD mice was not due to increased absorption or reduced excretion. If the mild increase in ALT and AST indicates hepatic damage in HCD mice, it was apparently of no consequence for plasma pharmacokinetics of LPS, even though it is excreted in bile [46].

Circulating LPS is carried by LPS-binding proteins and lipoproteins, particularly HDL [3,47-50]. Binding of LPS to lipoproteins inactivates LPS *in vitro *and *in vivo*, and reconstituted HDL is anti-inflammatory in animal and human endotoxemia [50-55]. Apolipoproteins themselves appear to be anti-inflammatory and can bind LPS [3,56]. The main apolipoprotein of normal HDL is ApoA1, but SAA and the LPS-interacting proteins, LBP and CD14, associate with HDL when they are induced [3,57]. In the present investigation, plasma levels of LBP and ApoA1 were not affected by HCD (Figure 8B and 8C). However, HCD increased plasma SAA as seen by others [20], and plasma CD14 was induced (Figure 8A).

Although the consequences of the increases in SAA and CD14 by HCD for a balance between pro- and anti-inflammatory actions of HDL are not known, a number of recent studies suggest that the effects of LPS should be reduced. SAA-HDL and normal HDL equally inhibited the induction of VCAM-1 by TNFα in endothelial cells [58]. However, SAA may also act indirectly to inhibit responses to LPS *in vivo*. For example, LBP, at concentrations normally found in plasma, antagonized the induction of TNFα by LPS in THP-1 cells *in vitro*. HDL purified from normal serum prevented this antagonism by LBP more effectively than HDL from critically ill subjects [59]. Since SAA, and probably SAA-HDL, was elevated in these patients, normal HDL may be a more effective antagonist of the anti-inflammatory effects of LBP than SAA-HDL. Thus, a net suppression of LPS activity might be expected when SAA is increased as it was in HCD mice. Although some CD14 is necessary for full activity of LPS at TLR4 receptors, elevated CD14 also antagonizes LPS [6]. If the net effect of elevated plasma SAA and CD14 is to inhibit LPS action, then the observed hypersensitivity of HCD mice may be due to a heightened response of target tissues. Increased hepatic TLR4 mRNA may again be significant in this respect. Further investigation is necessary to determine whether HCD alters the association of LPS with plasma lipoproteins, and whether changes in SAA, CD14 or other circulating factors affect the activity of LPS or its concentration in tissues.

## Conclusion

C57BL/6 mice developed hypercholesterolemia on a 4 wk HCD, accompanied by an inflammatory state with increased SAA and CD14. HCD caused an exaggerated response to LPS without affecting its plasma pharmacokinetics. Effects of HCD on liver, indicated by circulating liver enzymes and TLR4 mRNA, may contribute to the increased sensitivity to LPS. The results demonstrate that dietary cholesterol/fat can significantly affect the severity of the response to endotoxin.

## List of abbreviations

L-alanine:2-oxoglutarate aminotransferase, ALT

aspartateaminotransferase, AST

control diet, CD

high saturated fat and cholesterol diet, HCD

high density lipoprotein, HDL

inducible nitric oxide synthase, iNOS

interleukin-6, IL-6

interferon-γ, IFNγ

intraperitoneal, ip

lipopolysaccharide, LPS

lipopolysaccharide binding protein, LBP

nitric oxide, NO

phosphorylated at tyrosine 701, pY701

reverse transcription-polymerase chain reaction, RT-PCR

serum amyloid A, SAA

signal transducer and activator of transcription-1, STAT1

Toll-like receptor-4, TLR4

tumor necrosis factor-α, TNFα

vascular cell adhesion molecule -1, VCAM-1

## Competing interests

The author(s) declare that they have no competing interests.

## Authors' contributions

HH designed the study, and collected results in figures 1, 2, 3, 4, 5, 6, 7 and 9, measured cholesterol levels and serum amyloid, assessed liver histology, and drafted the manuscript. TL collected the results of figure 8 and contributed to the sections describing and discussing the results. RLS and JLR participated in processing mice and samples, western blotting and RT-PCR for VCAM-1 and iNOS, and contributed to the sections describing and discussing those results. DGH conceived and designed the study, aided in collection of results of figures 1, 2, 3, 4, 5, 6, 7 and 9, and processing of mice, and was the main author and editor of the manuscript. All authors read and approved the final manuscript.
